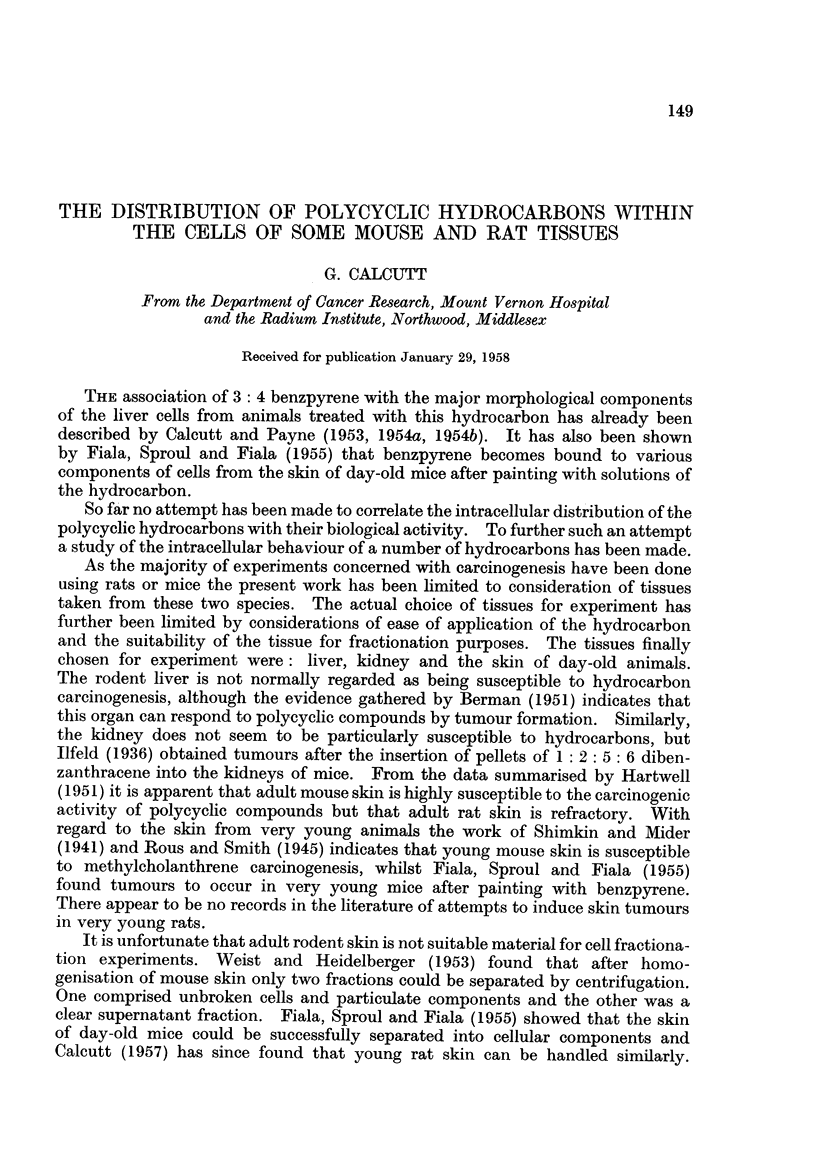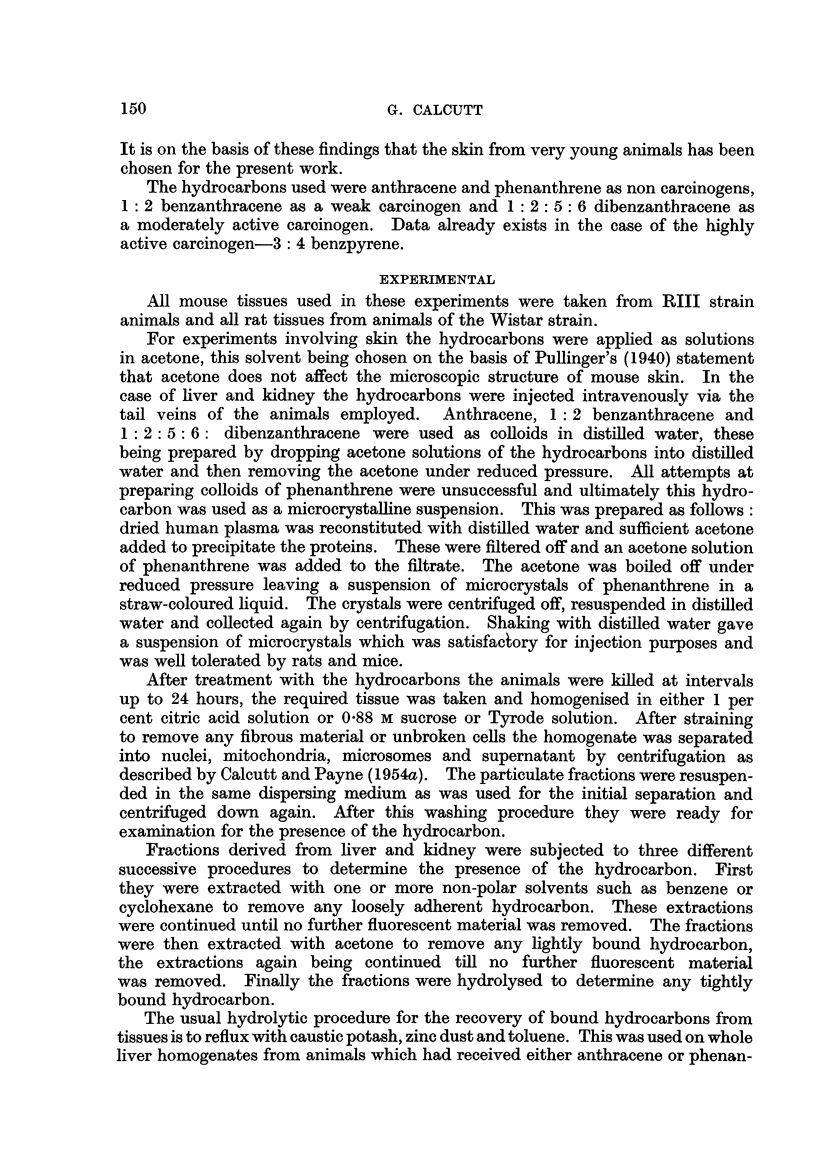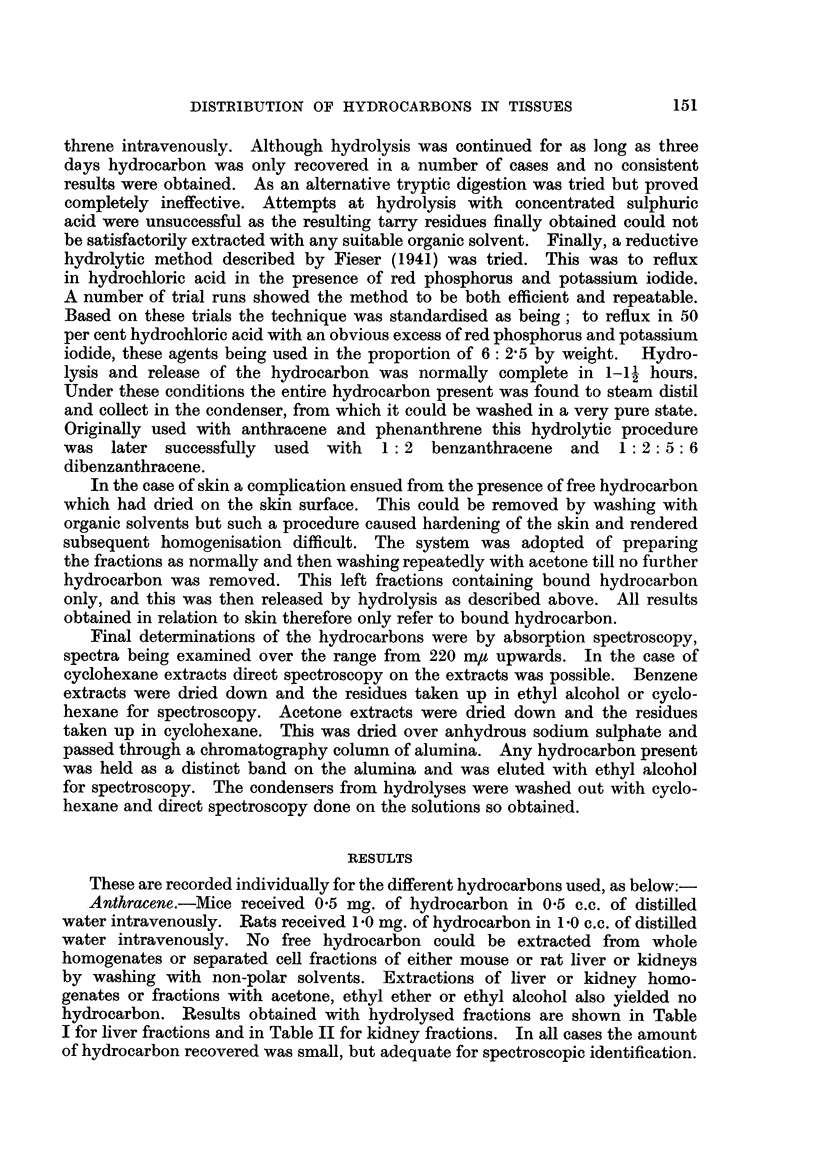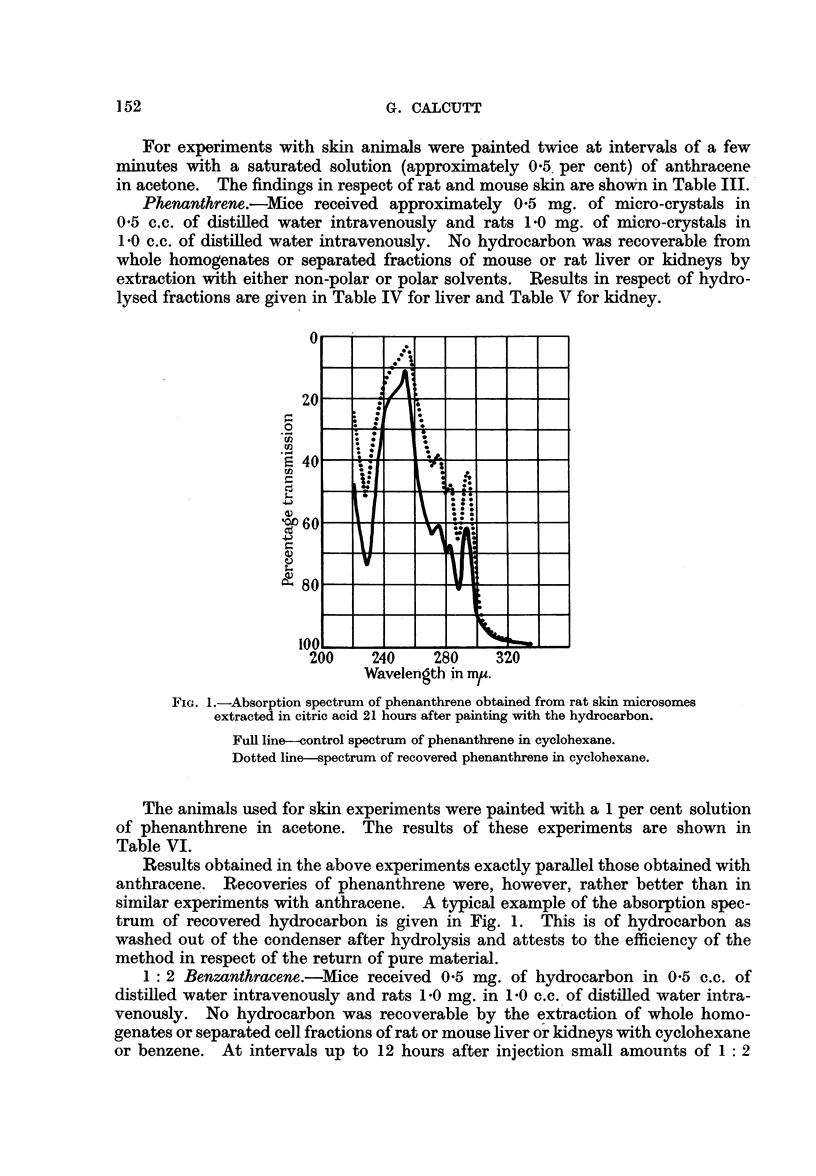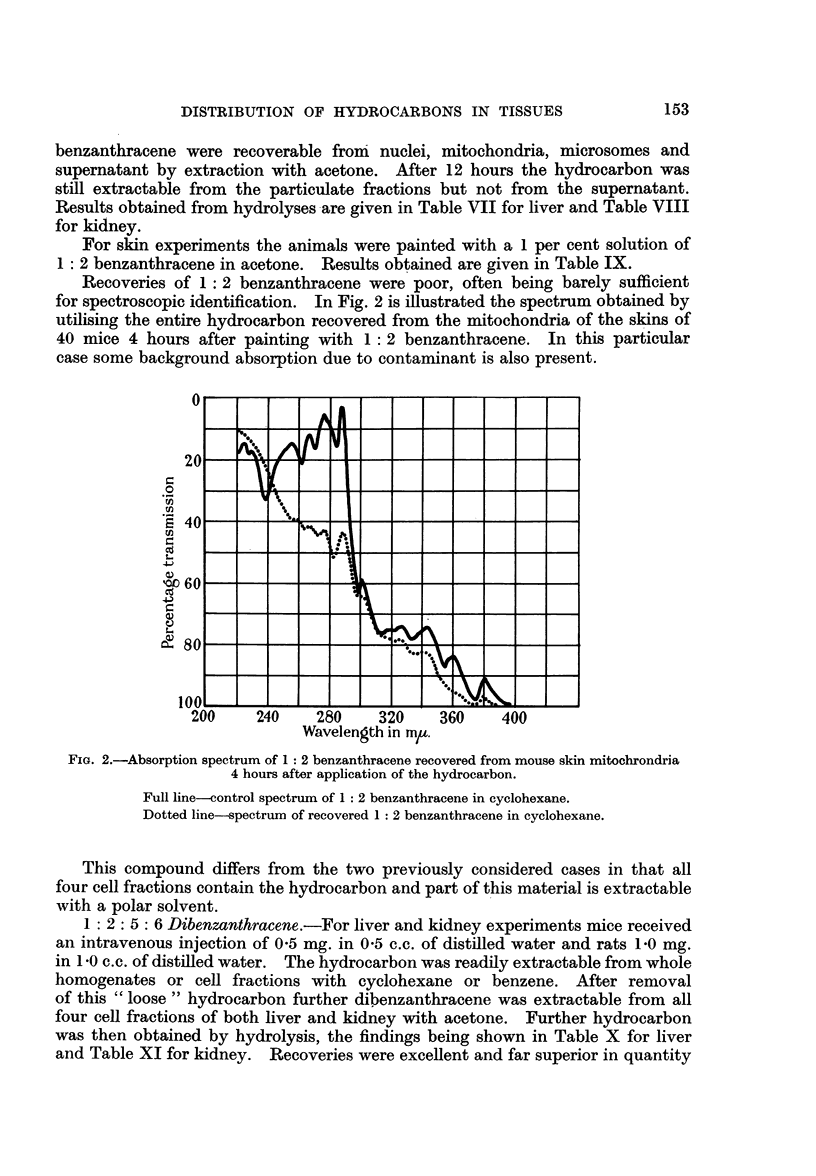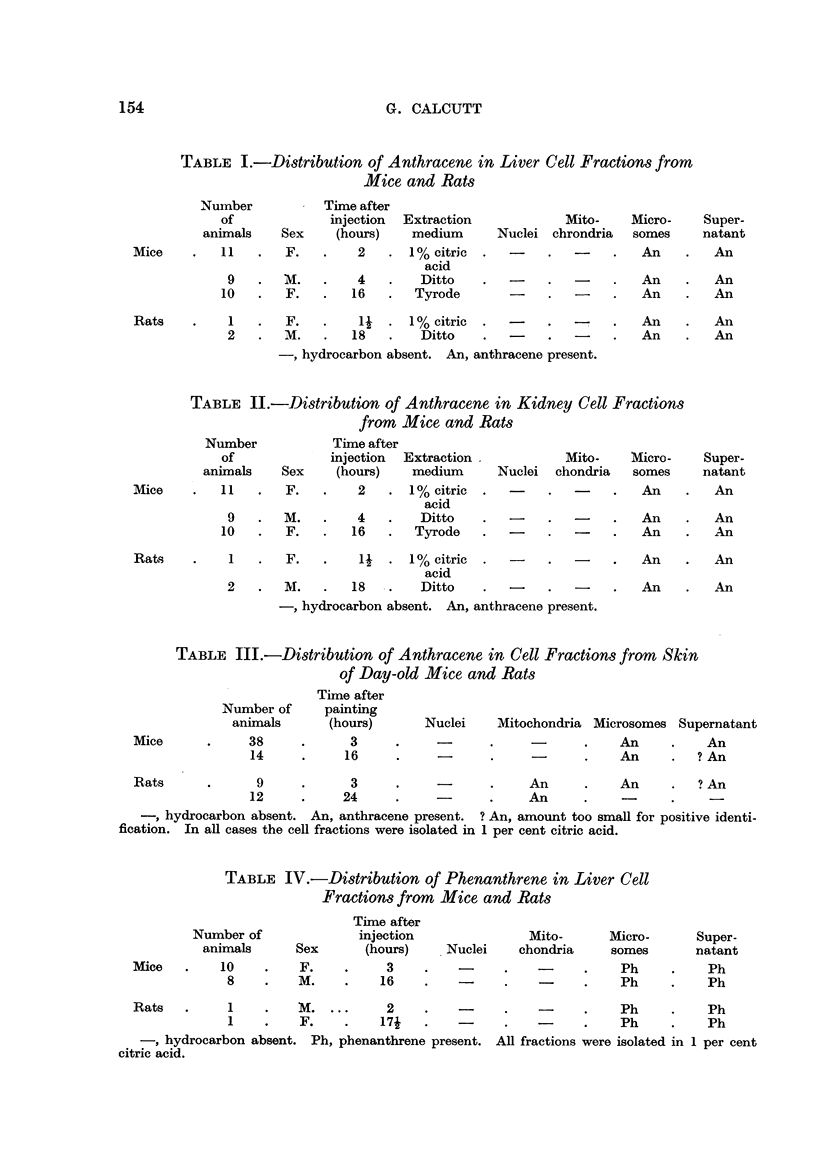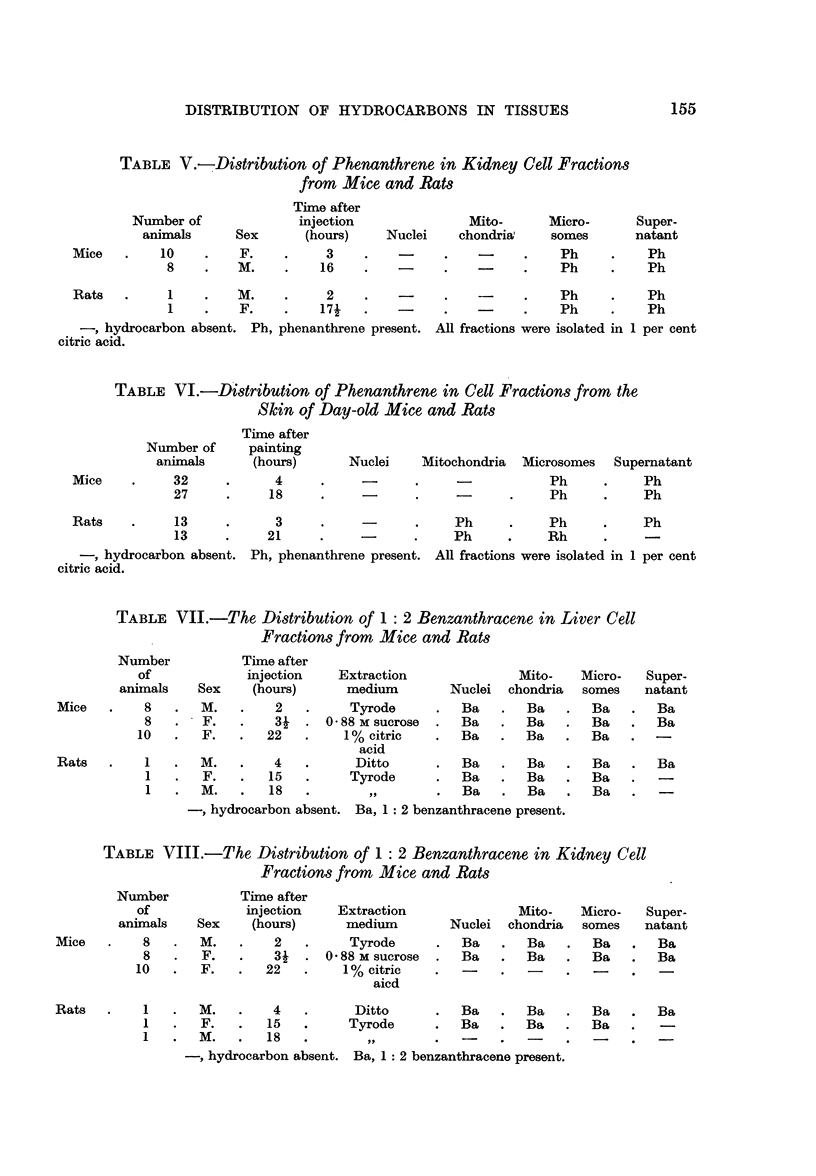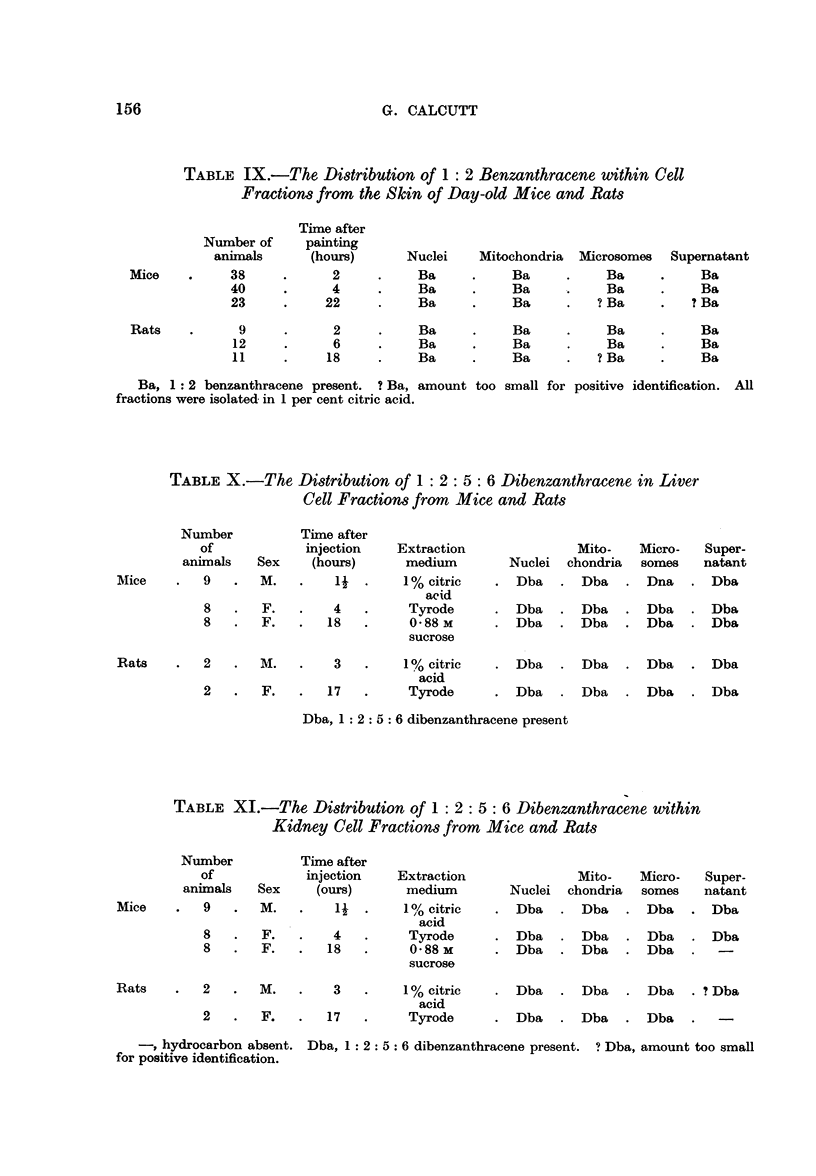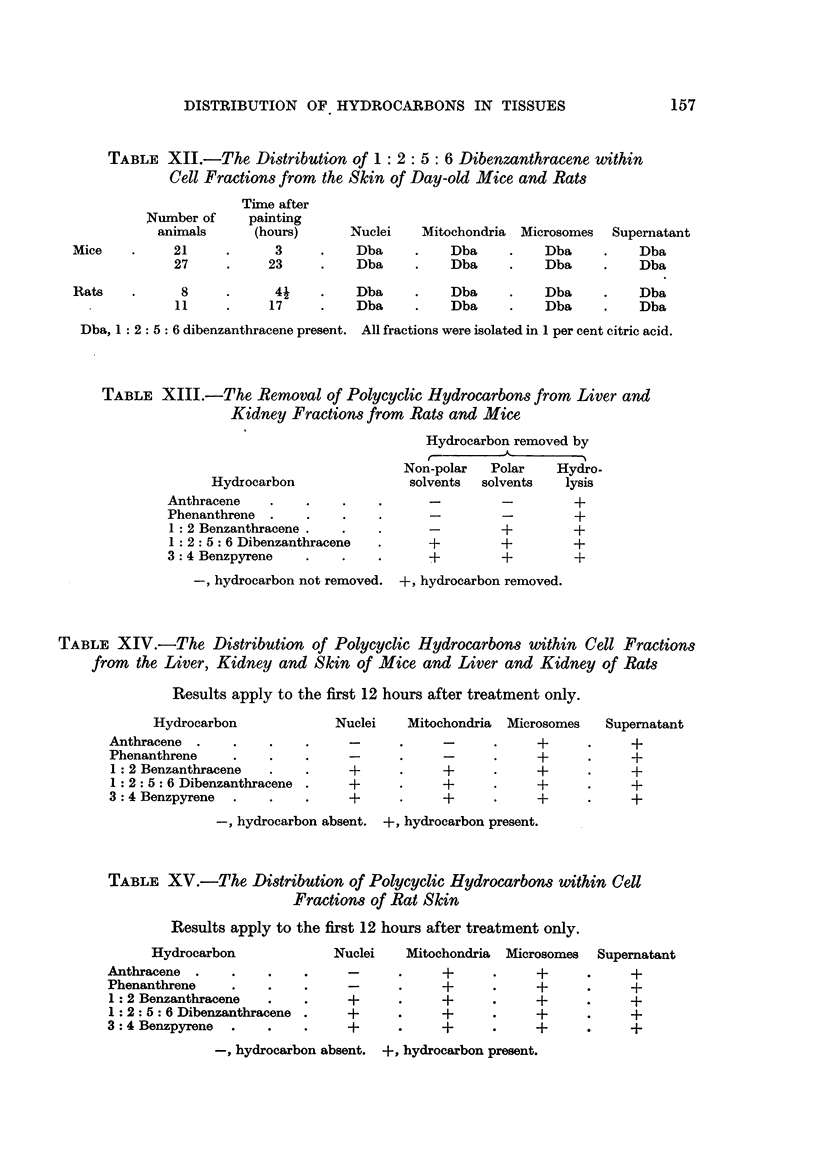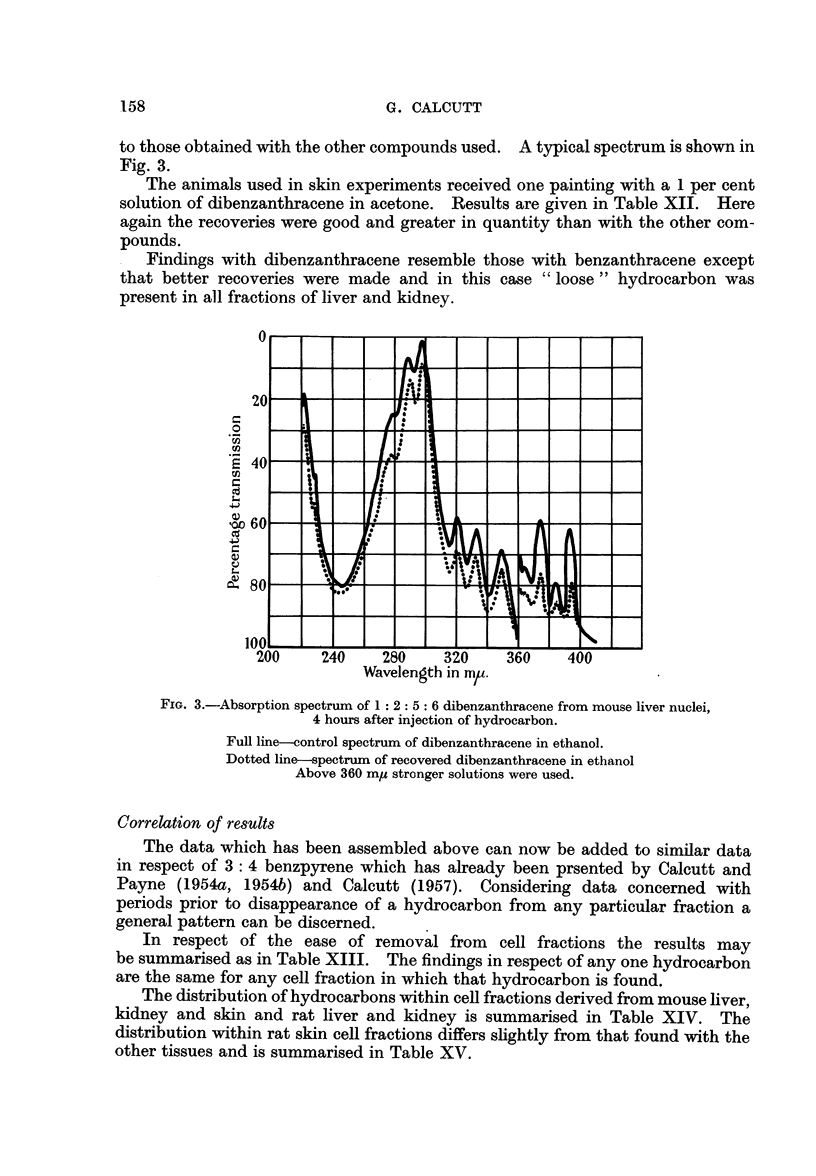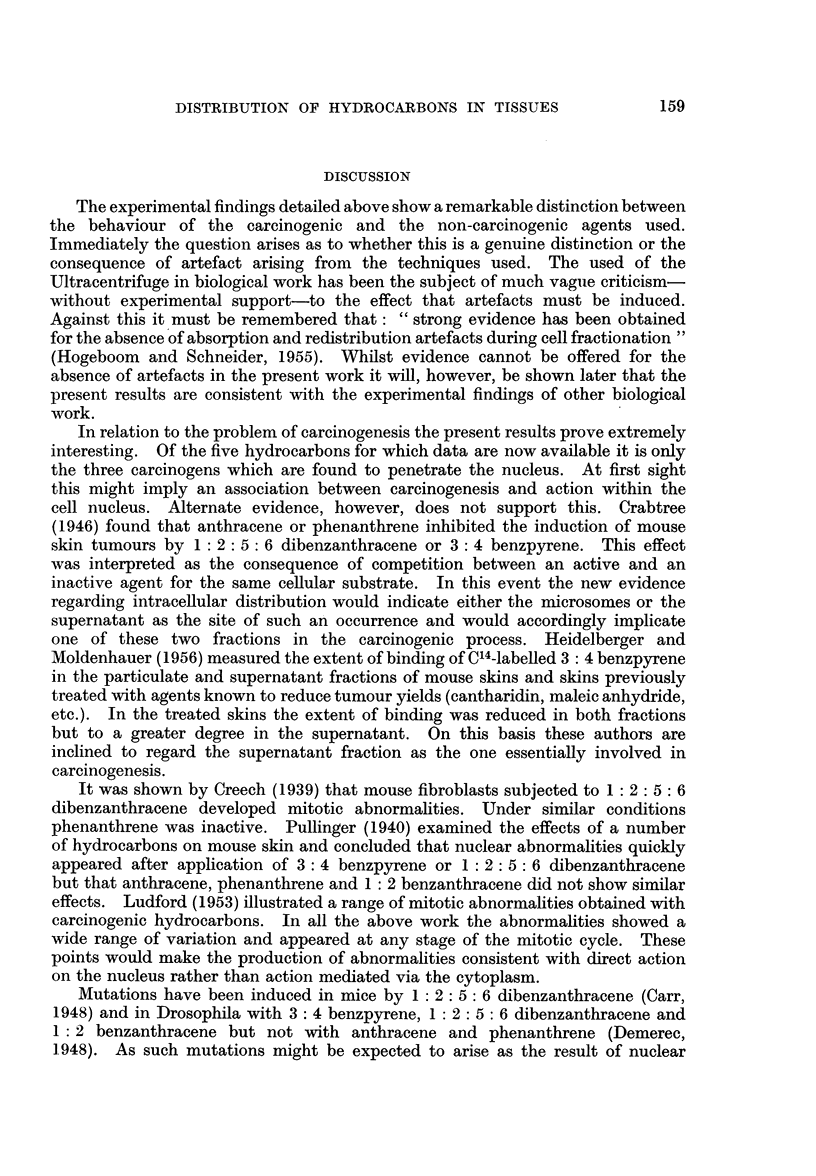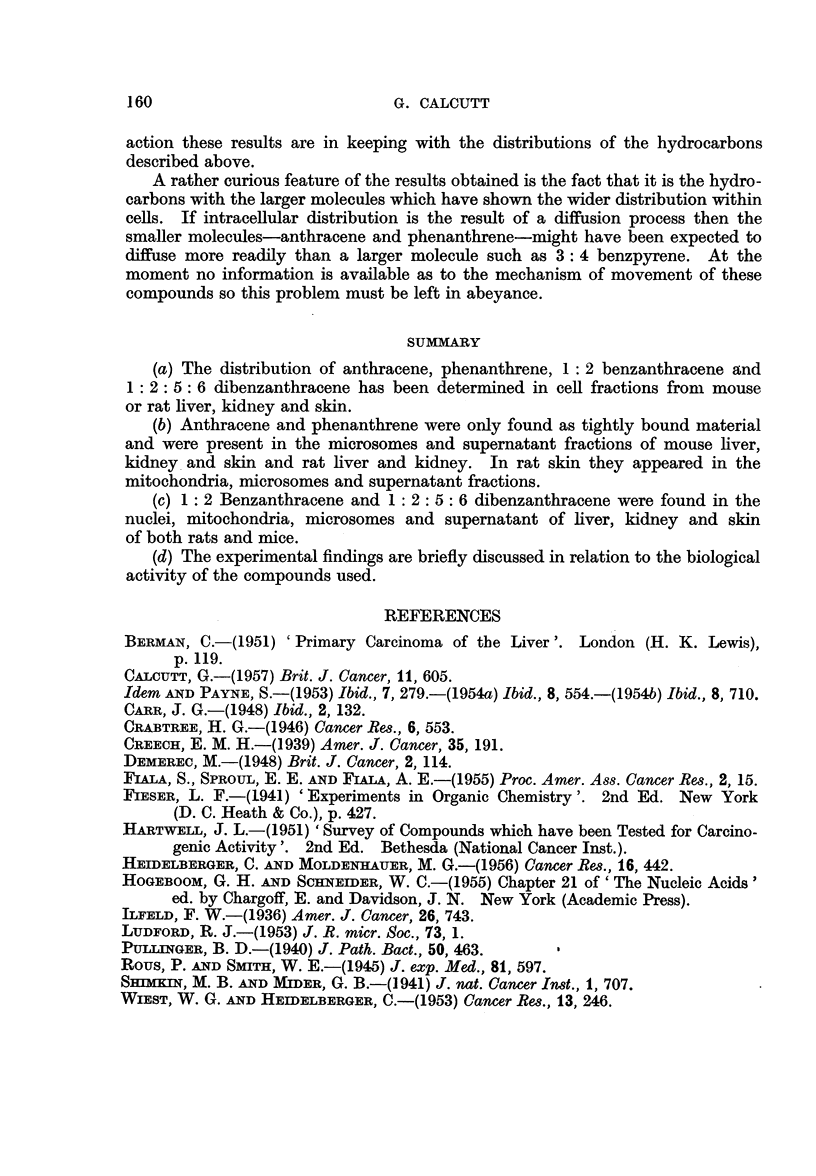# The Distribution of Polycyclic Hydrocarbons Within the Cells of some Mouse and Rat Tissues

**DOI:** 10.1038/bjc.1958.19

**Published:** 1958-03

**Authors:** G. Calcutt


### THE DISTRIBUTION OF POLYCYCLIC HYDROCARBONS WITHIN THE CELLS OF SOME MOUSE AND RAT TISSUES

G. CALCUTT

*From the Department of Cancer Research, Mount Vernon Hospital and the Radium Institute, Northwood, Middlesex*

Received for publication January 29, 1958

THE association of 3 : 4 benzpyrene with the major morphological components of the liver cells from animals treated with this hydrocarbon has already been described by Calcutt and Payne (1953, 1954*a*, 1954*b*). It has also been shown by Fiala, Sproul and Fiala (1955) that benzpyrene becomes bound to various components of cells from the skin of day-old mice after painting with solutions of the hydrocarbon.

So far no attempt has been made to correlate the intracellular distribution of the polycyclic hydrocarbons with their biological activity. To further such an attempt a study of the intracellular behaviour of a number of hydrocarbons has been made.

As the majority of experiments concerned with carcinogenesis have been done using rats or mice the present work has been limited to consideration of tissues taken from these two species. The actual choice of tissues for experiment has further been limited by considerations of ease of application of the hydrocarbon and the suitability of the tissue for fractionation purposes. The tissues finally chosen for experiment were : liver, kidney and the skin of day-old animals. The rodent liver is not normally regarded as being susceptible to hydrocarbon carcinogenesis, although the evidence gathered by Berman (1951) indicates that this organ can respond to polycyclic compounds by tumour formation. Similarly, the kidney does not seem to be particularly susceptible to hydrocarbons, but Ilfeld (1936) obtained tumours after the insertion of pellets of 1 : 2 : 5 : 6 dibenzanthracene into the kidneys of mice. From the data summarised by Hartwell (1951) it is apparent that adult mouse skin is highly susceptible to the carcinogenic activity of polycyclic compounds but that adult rat skin is refractory. With regard to the skin from very young animals the work of Shimkin and Mider (1941) and Rous and Smith (1945) indicates that young mouse skin is susceptible to methylcholanthrene carcinogenesis, whilst Fiala, Sproul and Fiala (1955) found tumours to occur in very young mice after painting with benzpyrene. There appear to be no records in the literature of attempts to induce skin tumours in very young rats.

It is unfortunate that adult rodent skin is not suitable material for cell fractionation experiments. Weist and Heidelberger (1953) found that after homogenisation of mouse skin only two fractions could be separated by centrifugation. One comprised unbroken cells and particulate components and the other was a clear supernatant fraction. Fiala, Sproul and Fiala (1955) showed that the skin of day-old mice could be successfully separated into cellular components and Calcutt (1957) has since found that young rat skin can be handled similarly.

It is on the basis of these findings that the skin from very young animals has been chosen for the present work.

The hydrocarbons used were anthracene and phenanthrene as non carcinogens, 1 : 2 benzanthracene as a weak carcinogen and 1 : 2 : 5 : 6 dibenzanthracene as a moderately active carcinogen. Data already exists in the case of the highly active carcinogen—3 : 4 benzpyrene.

### EXPERIMENTAL

All mouse tissues used in these experiments were taken from RIII strain animals and all rat tissues from animals of the Wistar strain.

For experiments involving skin the hydrocarbons were applied as solutions in acetone, this solvent being chosen on the basis of Pullinger's (1940) statement that acetone does not affect the microscopic structure of mouse skin. In the case of liver and kidney the hydrocarbons were injected intravenously via the tail veins of the animals employed. Anthracene, 1 : 2 benzanthracene and 1 : 2 : 5 : 6 : dibenzanthracene were used as colloids in distilled water, these being prepared by dropping acetone solutions of the hydrocarbons into distilled water and then removing the acetone under reduced pressure. All attempts at preparing colloids of phenanthrene were unsuccessful and ultimately this hydrocarbon was used as a microcrystalline suspension. This was prepared as follows : dried human plasma was reconstituted with distilled water and sufficient acetone added to precipitate the proteins. These were filtered off and an acetone solution of phenanthrene was added to the filtrate. The acetone was boiled off under reduced pressure leaving a suspension of microcrystals of phenanthrene in a straw-coloured liquid. The crystals were centrifuged off, resuspended in distilled water and collected again by centrifugation. Shaking with distilled water gave a suspension of microcrystals which was satisfactory for injection purposes and was well tolerated by rats and mice.

After treatment with the hydrocarbons the animals were killed at intervals up to 24 hours, the required tissue was taken and homogenised in either 1 per cent citric acid solution or 0·88 M sucrose or Tyrode solution. After straining to remove any fibrous material or unbroken cells the homogenate was separated into nuclei, mitochondria, microsomes and supernatant by centrifugation as described by Calcutt and Payne (1954*a*). The particulate fractions were resuspended in the same dispersing medium as was used for the initial separation and centrifuged down again. After this washing procedure they were ready for examination for the presence of the hydrocarbon.

Fractions derived from liver and kidney were subjected to three different successive procedures to determine the presence of the hydrocarbon. First they were extracted with one or more non-polar solvents such as benzene or cyclohexane to remove any loosely adherent hydrocarbon. These extractions were continued until no further fluorescent material was removed. The fractions were then extracted with acetone to remove any lightly bound hydrocarbon, the extractions again being continued till no further fluorescent material was removed. Finally the fractions were hydrolysed to determine any tightly bound hydrocarbon.

The usual hydrolytic procedure for the recovery of bound hydrocarbons from tissues is to reflux with caustic potash, zinc dust and toluene. This was used on whole liver homogenates from animals which had received either anthracene or phenanthrene intravenously. Although hydrolysis was continued for as long as three days hydrocarbon was only recovered in a number of cases and no consistent results were obtained. As an alternative tryptic digestion was tried but proved completely ineffective. Attempts at hydrolysis with concentrated sulphuric acid were unsuccessful as the resulting tarry residues finally obtained could not be satisfactorily extracted with any suitable organic solvent. Finally, a reductive hydrolytic method described by Fieser (1941) was tried. This was to reflux in hydrochloric acid in the presence of red phosphorus and potassium iodide. A number of trial runs showed the method to be both efficient and repeatable. Based on these trials the technique was standardised as being ; to reflux in 50 per cent hydrochloric acid with an obvious excess of red phosphorus and potassium iodide, these agents being used in the proportion of 6 : 2·5 by weight. Hydrolysis and release of the hydrocarbon was normally complete in 1–1½ hours. Under these conditions the entire hydrocarbon present was found to steam distil and collect in the condenser, from which it could be washed in a very pure state. Originally used with anthracene and phenanthrene this hydrolytic procedure was later successfully used with 1 : 2 benzanthracene and 1 : 2 : 5 : 6 dibenzanthracene.

In the case of skin a complication ensued from the presence of free hydrocarbon which had dried on the skin surface. This could be removed by washing with organic solvents but such a procedure caused hardening of the skin and rendered subsequent homogenisation difficult. The system was adopted of preparing the fractions as normally and then washing repeatedly with acetone till no further hydrocarbon was removed. This left fractions containing bound hydrocarbon only, and this was then released by hydrolysis as described above. All results obtained in relation to skin therefore only refer to bound hydrocarbon.

Final determinations of the hydrocarbons were by absorption spectroscopy, spectra being examined over the range from 220 mμ upwards. In the case of cyclohexane extracts direct spectroscopy on the extracts was possible. Benzene extracts were dried down and the residues taken up in ethyl alcohol or cyclohexane for spectroscopy. Acetone extracts were dried down and the residues taken up in cyclohexane. This was dried over anhydrous sodium sulphate and passed through a chromatography column of alumina. Any hydrocarbon present was held as a distinct band on the alumina and was eluted with ethyl alcohol for spectroscopy. The condensers from hydrolyses were washed out with cyclohexane and direct spectroscopy done on the solutions so obtained.

### RESULTS

These are recorded individually for the different hydrocarbons used, as below:—

*Anthracene.*—Mice received 0·5 mg. of hydrocarbon in 0·5 c.c. of distilled water intravenously. Rats received 1·0 mg. of hydrocarbon in 1·0 c.c. of distilled water intravenously. No free hydrocarbon could be extracted from whole homogenates or separated cell fractions of either mouse or rat liver or kidneys by washing with non-polar solvents. Extractions of liver or kidney homogenates or fractions with acetone, ethyl ether or ethyl alcohol also yielded no hydrocarbon. Results obtained with hydrolysed fractions are shown in Table I for liver fractions and in Table II for kidney fractions. In all cases the amount of hydrocarbon recovered was small, but adequate for spectroscopic identification.

For experiments with skin animals were painted twice at intervals of a few minutes with a saturated solution (approximately 0·5 per cent) of anthracene in acetone. The findings in respect of rat and mouse skin are shown in Table III.

*Phenanthrene.*—Mice received approximately 0·5 mg. of micro-crystals in 0·5 c.c. of distilled water intravenously and rats 1·0 mg. of micro-crystals in 1·0 c.c. of distilled water intravenously. No hydrocarbon was recoverable from whole homogenates or separated fractions of mouse or rat liver or kidneys by extraction with either non-polar or polar solvents. Results in respect of hydrolysed fractions are given in Table IV for liver and Table V for kidney.

FIG. 1.—Absorption spectrum of phenanthrene obtained from rat skin microsomes extracted in citric acid 21 hours after painting with the hydrocarbon.

Full line—control spectrum of phenanthrene in cyclohexane.

Dotted line—spectrum of recovered phenanthrene in cyclohexane.

The animals used for skin experiments were painted with a 1 per cent solution of phenanthrene in acetone. The results of these experiments are shown in Table VI.

Results obtained in the above experiments exactly parallel those obtained with anthracene. Recoveries of phenanthrene were, however, rather better than in similar experiments with anthracene. A typical example of the absorption spectrum of recovered hydrocarbon is given in Fig. 1. This is of hydrocarbon as washed out of the condenser after hydrolysis and attests to the efficiency of the method in respect of the return of pure material.

1 : 2 *Benzanthracene.*—Mice received 0·5 mg. of hydrocarbon in 0·5 c.c. of distilled water intravenously and rats 1·0 mg. in 1·0 c.c. of distilled water intravenously. No hydrocarbon was recoverable by the extraction of whole homogenates or separated cell fractions of rat or mouse liver or kidneys with cyclohexane or benzene. At intervals up to 12 hours after injection small amounts of 1 : 2

benzanthracene were recoverable from nuclei, mitochondria, microsomes and supernatant by extraction with acetone. After 12 hours the hydrocarbon was still extractable from the particulate fractions but not from the supernatant. Results obtained from hydrolyses are given in Table VII for liver and Table VIII for kidney.

For skin experiments the animals were painted with a 1 per cent solution of 1 : 2 benzanthracene in acetone. Results obtained are given in Table IX.

Recoveries of 1 : 2 benzanthracene were poor, often being barely sufficient for spectroscopic identification. In Fig. 2 is illustrated the spectrum obtained by utilising the entire hydrocarbon recovered from the mitochondria of the skins of 40 mice 4 hours after painting with 1 : 2 benzanthracene. In this particular case some background absorption due to contaminant is also present.

FIG. 2.—Absorption spectrum of 1 : 2 benzanthracene recovered from mouse skin mitochrondria 4 hours after application of the hydrocarbon.

Full line—control spectrum of 1 : 2 benzanthracene in cyclohexane.

Dotted line—spectrum of recovered 1 : 2 benzanthracene in cyclohexane.

This compound differs from the two previously considered cases in that all four cell fractions contain the hydrocarbon and part of this material is extractable with a polar solvent.

1 : 2 : 5 : 6 *Dibenzanthracene*.—For liver and kidney experiments mice received an intravenous injection of 0·5 mg. in 0·5 c.c. of distilled water and rats 1·0 mg. in 1·0 c.c. of distilled water. The hydrocarbon was readily extractable from whole homogenates or cell fractions with cyclohexane or benzene. After removal of this " loose " hydrocarbon further dibenzanthracene was extractable from all four cell fractions of both liver and kidney with acetone. Further hydrocarbon was then obtained by hydrolysis, the findings being shown in Table X for liver and Table XI for kidney. Recoveries were excellent and far superior in quantity

TABLE I.—*Distribution of Anthracene in Liver Cell Fractions from Mice and Rats*

| | Number of animals | Sex | Time after injection (hours) | Extraction medium | Nuclei | Mito-chrondria | Micro-somes | Super-natant |
|---|---|---|---|---|---|---|---|---|
| Mice | 11 | F. | 2 | 1% citric acid | — | — | An | An |
| | 9 | M. | 4 | Ditto | — | — | An | An |
| | 10 | F. | 16 | Tyrode | — | — | An | An |
| Rats | 1 | F. | 1½ | 1% citric | — | — | An | An |
| | 2 | M. | 18 | Ditto | — | — | An | An |

—, hydrocarbon absent. An, anthracene present.

TABLE II.—*Distribution of Anthracene in Kidney Cell Fractions from Mice and Rats*

| | Number of animals | Sex | Time after injection (hours) | Extraction medium | Nuclei | Mito-chondria | Micro-somes | Super-natant |
|---|---|---|---|---|---|---|---|---|
| Mice | 11 | F. | 2 | 1% citric acid | — | — | An | An |
| | 9 | M. | 4 | Ditto | — | — | An | An |
| | 10 | F. | 16 | Tyrode | — | — | An | An |
| Rats | 1 | F. | 1½ | 1% citric acid | — | — | An | An |
| | 2 | M. | 18 | Ditto | — | — | An | An |

—, hydrocarbon absent. An, anthracene present.

TABLE III.—*Distribution of Anthracene in Cell Fractions from Skin of Day-old Mice and Rats*

| | Number of animals | Time after painting (hours) | Nuclei | Mitochondria | Microsomes | Supernatant |
|---|---|---|---|---|---|---|
| Mice | 38 | 3 | — | — | An | An |
| | 14 | 16 | — | — | An | ? An |
| Rats | 9 | 3 | — | An | An | ? An |
| | 12 | 24 | — | An | — | — |

—, hydrocarbon absent. An, anthracene present. ? An, amount too small for positive identification. In all cases the cell fractions were isolated in 1 per cent citric acid.

TABLE IV.—*Distribution of Phenanthrene in Liver Cell Fractions from Mice and Rats*

| | Number of animals | Sex | Time after injection (hours) | Nuclei | Mito-chondria | Micro-somes | Super-natant |
|---|---|---|---|---|---|---|---|
| Mice | 10 | F. | 3 | — | — | Ph | Ph |
| | 8 | M. | 16 | — | — | Ph | Ph |
| Rats | 1 | M. | 2 | — | — | Ph | Ph |
| | 1 | F. | 17½ | — | — | Ph | Ph |

—, hydrocarbon absent. Ph, phenanthrene present. All fractions were isolated in 1 per cent citric acid.

TABLE V.—*Distribution of Phenanthrene in Kidney Cell Fractions from Mice and Rats*

| | Number of animals | Sex | Time after injection (hours) | Nuclei | Mito-chondria | Micro-somes | Super-natant |
|---|---|---|---|---|---|---|---|
| Mice | 10 | F. | 3 | — | — | Ph | Ph |
| | 8 | M. | 16 | — | — | Ph | Ph |
| Rats | 1 | M. | 2 | — | — | Ph | Ph |
| | 1 | F. | 17½ | — | — | Ph | Ph |

—, hydrocarbon absent. Ph, phenanthrene present. All fractions were isolated in 1 per cent citric acid.

TABLE VI.—*Distribution of Phenanthrene in Cell Fractions from the Skin of Day-old Mice and Rats*

| | Number of animals | Time after painting (hours) | Nuclei | Mitochondria | Microsomes | Supernatant |
|---|---|---|---|---|---|---|
| Mice | 32 | 4 | — | — | Ph | Ph |
| | 27 | 18 | — | — | Ph | Ph |
| Rats | 13 | 3 | — | Ph | Ph | Ph |
| | 13 | 21 | — | Ph | Rh | — |

—, hydrocarbon absent. Ph, phenanthrene present. All fractions were isolated in 1 per cent citric acid.

TABLE VII.—*The Distribution of* 1 : 2 *Benzanthracene in Liver Cell Fractions from Mice and Rats*

| | Number of animals | Sex | Time after injection (hours) | Extraction medium | Nuclei | Mito-chondria | Micro-somes | Super-natant |
|---|---|---|---|---|---|---|---|---|
| Mice | 8 | M. | 2 | Tyrode | Ba | Ba | Ba | Ba |
| | 8 | F. | 3½ | 0·88 M sucrose | Ba | Ba | Ba | Ba |
| | 10 | F. | 22 | 1% citric acid | Ba | Ba | Ba | — |
| Rats | 1 | M. | 4 | Ditto | Ba | Ba | Ba | Ba |
| | 1 | F. | 15 | Tyrode | Ba | Ba | Ba | — |
| | 1 | M. | 18 | ,, | Ba | Ba | Ba | — |

—, hydrocarbon absent. Ba, 1 : 2 benzanthracene present.

TABLE VIII.—*The Distribution of* 1 : 2 *Benzanthracene in Kidney Cell Fractions from Mice and Rats*

| | Number of animals | Sex | Time after injection (hours) | Extraction medium | Nuclei | Mito-chondria | Micro-somes | Super-natant |
|---|---|---|---|---|---|---|---|---|
| Mice | 8 | M. | 2 | Tyrode | Ba | Ba | Ba | Ba |
| | 8 | F. | 3½ | 0·88 M sucrose | Ba | Ba | Ba | Ba |
| | 10 | F. | 22 | 1% citric aicd | — | — | — | — |
| Rats | 1 | M. | 4 | Ditto | Ba | Ba | Ba | Ba |
| | 1 | F. | 15 | Tyrode | Ba | Ba | Ba | — |
| | 1 | M. | 18 | ,, | — | — | — | — |

—, hydrocarbon absent. Ba, 1 : 2 benzanthracene present.

TABLE IX.—*The Distribution of* 1 : 2 *Benzanthracene within Cell Fractions from the Skin of Day-old Mice and Rats*

| | Number of animals | Time after painting (hours) | Nuclei | Mitochondria | Microsomes | Supernatant |
|---|---|---|---|---|---|---|
| Mice | 38 | 2 | Ba | Ba | Ba | Ba |
| | 40 | 4 | Ba | Ba | Ba | Ba |
| | 23 | 22 | Ba | Ba | ? Ba | ? Ba |
| Rats | 9 | 2 | Ba | Ba | Ba | Ba |
| | 12 | 6 | Ba | Ba | Ba | Ba |
| | 11 | 18 | Ba | Ba | ? Ba | Ba |

Ba, 1 : 2 benzanthracene present. ? Ba, amount too small for positive identification. All fractions were isolated in 1 per cent citric acid.

TABLE X.—*The Distribution of* 1 : 2 : 5 : 6 *Dibenzanthracene in Liver Cell Fractions from Mice and Rats*

| | Number of animals | Sex | Time after injection (hours) | Extraction medium | Nuclei | Mito-chondria | Micro-somes | Super-natant |
|---|---|---|---|---|---|---|---|---|
| Mice | 9 | M. | 1½ | 1% citric acid | Dba | Dba | Dna | Dba |
| | 8 | F. | 4 | Tyrode | Dba | Dba | Dba | Dba |
| | 8 | F. | 18 | 0·88 M sucrose | Dba | Dba | Dba | Dba |
| Rats | 2 | M. | 3 | 1% citric acid | Dba | Dba | Dba | Dba |
| | 2 | F. | 17 | Tyrode | Dba | Dba | Dba | Dba |

Dba, 1 : 2 : 5 : 6 dibenzanthracene present

TABLE XI.—*The Distribution of* 1 : 2 : 5 : 6 *Dibenzanthracene within Kidney Cell Fractions from Mice and Rats*

| | Number of animals | Sex | Time after injection (ours) | Extraction medium | Nuclei | Mito-chondria | Micro-somes | Super-natant |
|---|---|---|---|---|---|---|---|---|
| Mice | 9 | M. | 1½ | 1% citric acid | Dba | Dba | Dba | Dba |
| | 8 | F. | 4 | Tyrode | Dba | Dba | Dba | Dba |
| | 8 | F. | 18 | 0·88 M sucrose | Dba | Dba | Dba | — |
| Rats | 2 | M. | 3 | 1% citric acid | Dba | Dba | Dba | ? Dba |
| | 2 | F. | 17 | Tyrode | Dba | Dba | Dba | — |

—, hydrocarbon absent. Dba, 1 : 2 : 5 : 6 dibenzanthracene present. ? Dba, amount too small for positive identification.

TABLE XII.—*The Distribution of* 1 : 2 : 5 : 6 *Dibenzanthracene within Cell Fractions from the Skin of Day-old Mice and Rats*

| | Number of animals | Time after painting (hours) | Nuclei | Mitochondria | Microsomes | Supernatant |
|---|---|---|---|---|---|---|
| Mice | 21 | 3 | Dba | Dba | Dba | Dba |
| | 27 | 23 | Dba | Dba | Dba | Dba |
| Rats | 8 | 4½ | Dba | Dba | Dba | Dba |
| | 11 | 17 | Dba | Dba | Dba | Dba |

Dba, 1 : 2 : 5 : 6 dibenzanthracene present. All fractions were isolated in 1 per cent citric acid.

TABLE XIII.—*The Removal of Polycyclic Hydrocarbons from Liver and Kidney Fractions from Rats and Mice*

| Hydrocarbon | Hydrocarbon removed by: Non-polar solvents | Polar solvents | Hydrolysis |
|---|---|---|---|
| Anthracene | — | — | + |
| Phenanthrene | — | — | + |
| 1 : 2 Benzanthracene | — | + | + |
| 1 : 2 : 5 : 6 Dibenzanthracene | + | + | + |
| 3 : 4 Benzpyrene | + | + | + |

—, hydrocarbon not removed. +, hydrocarbon removed.

TABLE XIV.—*The Distribution of Polycyclic Hydrocarbons within Cell Fractions from the Liver, Kidney and Skin of Mice and Liver and Kidney of Rats*

Results apply to the first 12 hours after treatment only.

| Hydrocarbon | Nuclei | Mitochondria | Microsomes | Supernatant |
|---|---|---|---|---|
| Anthracene | — | — | + | + |
| Phenanthrene | — | — | + | + |
| 1 : 2 Benzanthracene | + | + | + | + |
| 1 : 2 : 5 : 6 Dibenzanthracene | + | + | + | + |
| 3 : 4 Benzpyrene | + | + | + | + |

—, hydrocarbon absent. +, hydrocarbon present.

TABLE XV.—*The Distribution of Polycyclic Hydrocarbons within Cell Fractions of Rat Skin*

Results apply to the first 12 hours after treatment only.

| Hydrocarbon | Nuclei | Mitochondria | Microsomes | Supernatant |
|---|---|---|---|---|
| Anthracene | — | + | + | + |
| Phenanthrene | — | + | + | + |
| 1 : 2 Benzanthracene | + | + | + | + |
| 1 : 2 : 5 : 6 Dibenzanthracene | + | + | + | + |
| 3 : 4 Benzpyrene | + | + | + | + |

—, hydrocarbon absent. +, hydrocarbon present.

to those obtained with the other compounds used. A typical spectrum is shown in Fig. 3.

The animals used in skin experiments received one painting with a 1 per cent solution of dibenzanthracene in acetone. Results are given in Table XII. Here again the recoveries were good and greater in quantity than with the other compounds.

Findings with dibenzanthracene resemble those with benzanthracene except that better recoveries were made and in this case "loose" hydrocarbon was present in all fractions of liver and kidney.

FIG. 3.—Absorption spectrum of 1 : 2 : 5 : 6 dibenzanthracene from mouse liver nuclei, 4 hours after injection of hydrocarbon.

Full line—control spectrum of dibenzanthracene in ethanol.
Dotted line—spectrum of recovered dibenzanthracene in ethanol
Above 360 mμ stronger solutions were used.

*Correlation of results*

The data which has been assembled above can now be added to similar data in respect of 3 : 4 benzpyrene which has already been prsented by Calcutt and Payne (1954*a*, 1954*b*) and Calcutt (1957). Considering data concerned with periods prior to disappearance of a hydrocarbon from any particular fraction a general pattern can be discerned.

In respect of the ease of removal from cell fractions the results may be summarised as in Table XIII. The findings in respect of any one hydrocarbon are the same for any cell fraction in which that hydrocarbon is found.

The distribution of hydrocarbons within cell fractions derived from mouse liver, kidney and skin and rat liver and kidney is summarised in Table XIV. The distribution within rat skin cell fractions differs slightly from that found with the other tissues and is summarised in Table XV.

### DISCUSSION

The experimental findings detailed above show a remarkable distinction between the behaviour of the carcinogenic and the non-carcinogenic agents used. Immediately the question arises as to whether this is a genuine distinction or the consequence of artefact arising from the techniques used. The used of the Ultracentrifuge in biological work has been the subject of much vague criticism—without experimental support—to the effect that artefacts must be induced. Against this it must be remembered that : " strong evidence has been obtained for the absence of absorption and redistribution artefacts during cell fractionation " (Hogeboom and Schneider, 1955). Whilst evidence cannot be offered for the absence of artefacts in the present work it will, however, be shown later that the present results are consistent with the experimental findings of other biological work.

In relation to the problem of carcinogenesis the present results prove extremely interesting. Of the five hydrocarbons for which data are now available it is only the three carcinogens which are found to penetrate the nucleus. At first sight this might imply an association between carcinogenesis and action within the cell nucleus. Alternate evidence, however, does not support this. Crabtree (1946) found that anthracene or phenanthrene inhibited the induction of mouse skin tumours by 1 : 2 : 5 : 6 dibenzanthracene or 3 : 4 benzpyrene. This effect was interpreted as the consequence of competition between an active and an inactive agent for the same cellular substrate. In this event the new evidence regarding intracellular distribution would indicate either the microsomes or the supernatant as the site of such an occurrence and would accordingly implicate one of these two fractions in the carcinogenic process. Heidelberger and Moldenhauer (1956) measured the extent of binding of $C^{14}$-labelled 3 : 4 benzpyrene in the particulate and supernatant fractions of mouse skins and skins previously treated with agents known to reduce tumour yields (cantharidin, maleic anhydride, etc.). In the treated skins the extent of binding was reduced in both fractions but to a greater degree in the supernatant. On this basis these authors are inclined to regard the supernatant fraction as the one essentially involved in carcinogenesis.

It was shown by Creech (1939) that mouse fibroblasts subjected to 1 : 2 : 5 : 6 dibenzanthracene developed mitotic abnormalities. Under similar conditions phenanthrene was inactive. Pullinger (1940) examined the effects of a number of hydrocarbons on mouse skin and concluded that nuclear abnormalities quickly appeared after application of 3 : 4 benzpyrene or 1 : 2 : 5 : 6 dibenzanthracene but that anthracene, phenanthrene and 1 : 2 benzanthracene did not show similar effects. Ludford (1953) illustrated a range of mitotic abnormalities obtained with carcinogenic hydrocarbons. In all the above work the abnormalities showed a wide range of variation and appeared at any stage of the mitotic cycle. These points would make the production of abnormalities consistent with direct action on the nucleus rather than action mediated via the cytoplasm.

Mutations have been induced in mice by 1 : 2 : 5 : 6 dibenzanthracene (Carr, 1948) and in Drosophila with 3 : 4 benzpyrene, 1 : 2 : 5 : 6 dibenzanthracene and 1 : 2 benzanthracene but not with anthracene and phenanthrene (Demerec, 1948). As such mutations might be expected to arise as the result of nuclear action these results are in keeping with the distributions of the hydrocarbons described above.

A rather curious feature of the results obtained is the fact that it is the hydrocarbons with the larger molecules which have shown the wider distribution within cells. If intracellular distribution is the result of a diffusion process then the smaller molecules—anthracene and phenanthrene—might have been expected to diffuse more readily than a larger molecule such as 3 : 4 benzpyrene. At the moment no information is available as to the mechanism of movement of these compounds so this problem must be left in abeyance.

### SUMMARY

(*a*) The distribution of anthracene, phenanthrene, 1 : 2 benzanthracene and 1 : 2 : 5 : 6 dibenzanthracene has been determined in cell fractions from mouse or rat liver, kidney and skin.

(*b*) Anthracene and phenanthrene were only found as tightly bound material and were present in the microsomes and supernatant fractions of mouse liver, kidney and skin and rat liver and kidney. In rat skin they appeared in the mitochondria, microsomes and supernatant fractions.

(*c*) 1 : 2 Benzanthracene and 1 : 2 : 5 : 6 dibenzanthracene were found in the nuclei, mitochondria, microsomes and supernatant of liver, kidney and skin of both rats and mice.

(*d*) The experimental findings are briefly discussed in relation to the biological activity of the compounds used.

### REFERENCES

Berman, C.—(1951) 'Primary Carcinoma of the Liver'. London (H. K. Lewis), p. 119.
Calcutt, G.—(1957) *Brit. J. Cancer*, **11**, 605.
*Idem* and Payne, S.—(1953) *Ibid.*, **7**, 279.—(1954*a*) *Ibid.*, **8**, 554.—(1954*b*) *Ibid.*, **8**, 710.
Carr, J. G.—(1948) *Ibid.*, **2**, 132.
Crabtree, H. G.—(1946) *Cancer Res.*, **6**, 553.
Creech, E. M. H.—(1939) *Amer. J. Cancer*, **35**, 191.
Demerec, M.—(1948) *Brit. J. Cancer*, **2**, 114.
Fiala, S., Sproul, E. E. and Fiala, A. E.—(1955) *Proc. Amer. Ass. Cancer Res.*, **2**, 15.
Fieser, L. F.—(1941) 'Experiments in Organic Chemistry'. 2nd Ed. New York (D. C. Heath & Co.), p. 427.
Hartwell, J. L.—(1951) 'Survey of Compounds which have been Tested for Carcinogenic Activity'. 2nd Ed. Bethesda (National Cancer Inst.).
Heidelberger, C. and Moldenhauer, M. G.—(1956) *Cancer Res.*, **16**, 442.
Hogeboom, G. H. and Schneider, W. C.—(1955) Chapter 21 of 'The Nucleic Acids' ed. by Chargoff, E. and Davidson, J. N. New York (Academic Press).
Ilfeld, F. W.—(1936) *Amer. J. Cancer*, **26**, 743.
Ludford, R. J.—(1953) *J. R. micr. Soc.*, **73**, 1.
Pullinger, B. D.—(1940) *J. Path. Bact.*, **50**, 463.
Rous, P. and Smith, W. E.—(1945) *J. exp. Med.*, **81**, 597.
Shimkin, M. B. and Mider, G. B.—(1941) *J. nat. Cancer Inst.*, **1**, 707.
Wiest, W. G. and Heidelberger, C.—(1953) *Cancer Res.*, **13**, 246.